# *N*-[(*E*)-1,3-Benzodioxol-5-yl­methyl­idene]-4-methyl­aniline

**DOI:** 10.1107/S1600536810048038

**Published:** 2010-11-24

**Authors:** M. Nawaz Tahir, Hazoor Ahmad Shad, Muhammad Naeem Khan, Riaz H. Tariq

**Affiliations:** a Department of Physics, University of Sargodha, Sargodha, Pakistan; b Department of Chemistry, Govt. M. D. College, Toba Tek Singh, Punjab, Pakistan; c Applied Chemistry Research Center, PCSIR Laboratories Complex, Lahore 54600, Pakistan; d Institute of Chemical and Pharmaceutical Sciences, The University of Faisalabad, Faisalabad, Pakistan

## Abstract

The two symmetry-independent mol­ecules in the asymmetric unit of the title compound, C_15_H_13_NO_2_, differ in conformation, with the virtually planar 4-methyl­aniline (r.m.s. deviations of 0.0511 and 0.0082 Å) and piperonal groups (r.m.s. deviations of 0.0241 and 0.0486 Å) forming dihedral angles of 19.40 (5) and 42.90 (6)°. In the crystal, mol­ecules are linked by C—H⋯O and C—H⋯π inter­actions. The H atoms of the two methyl groups are disordered over two sets of sites of equal occupancy.

## Related literature

For background to our ongoing project on the synthesis of various Schiff bases of piperonal and then their metal complexation and for a related structure, see: Tahir *et al.* (2010 ▶).
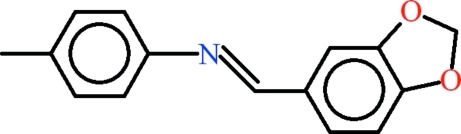


## Experimental

### 

#### Crystal data

C_15_H_13_NO_2_
*M* *_r_* = 239.26
Triclinic, 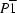

*a* = 10.6914 (4) Å
*b* = 10.7680 (3) Å
*c* = 13.3332 (5) Å
α = 89.443 (2)°
β = 67.112 (2)°
γ = 62.534 (1)°
*V* = 1227.41 (8) Å^3^
*Z* = 4
Mo *K*α radiation
μ = 0.09 mm^−1^
*T* = 296 K
0.32 × 0.22 × 0.18 mm

#### Data collection

Bruker Kappa APEXII CCD diffractometer
Absorption correction: multi-scan (*SADABS*; Bruker, 2005 ▶) *T*_min_ = 0.980, *T*_max_ = 0.988
18069 measured reflections
4417 independent reflections
3294 reflections with *I* > 2σ(*I*)
*R*_int_ = 0.026

#### Refinement

*R*[*F* ^2^ > 2σ(*F* ^2^)] = 0.039
*wR*(*F* ^2^) = 0.110
*S* = 1.03
4417 reflections
323 parameters
H-atom parameters constrained
Δρ_max_ = 0.17 e Å^−3^
Δρ_min_ = −0.15 e Å^−3^

### 

Data collection: *APEX2* (Bruker, 2009 ▶); cell refinement: *SAINT* (Bruker, 2009 ▶); data reduction: *SAINT*; program(s) used to solve structure: *SHELXS97* (Sheldrick, 2008 ▶); program(s) used to refine structure: *SHELXL97* (Sheldrick, 2008 ▶); molecular graphics: *ORTEP-3 for Windows* (Farrugia, 1997 ▶) and *PLATON* (Spek, 2009 ▶); software used to prepare material for publication: *WinGX* (Farrugia, 1999 ▶) and *PLATON*.

## Supplementary Material

Crystal structure: contains datablocks global, I. DOI: 10.1107/S1600536810048038/gk2324sup1.cif

Structure factors: contains datablocks I. DOI: 10.1107/S1600536810048038/gk2324Isup2.hkl

Additional supplementary materials:  crystallographic information; 3D view; checkCIF report

## Figures and Tables

**Table 1:** Hydrogen-bond geometry (Å, °) *Cg*3, *Cg*5 and *Cg*6 are the centroids of the C9–C14, C16–C21 and C24–C29 rings, respectively.

*D*—H⋯*A*	*D*—H	H⋯*A*	*D*⋯*A*	*D*—H⋯*A*
C28—H28⋯O2^i^	0.93	2.59	3.447 (2)	153
C17—H17⋯*Cg*3^ii^	0.93	2.73	3.5306 (16)	145
C22—H22*B*⋯*Cg*5^ii^	0.96	2.86	3.5480 (18)	130
C22—H22*D*⋯*Cg*5^ii^	0.96	2.71	3.5480 (18)	146
C22—H22*E*⋯*Cg*6^iii^	0.96	2.91	3.8610 (18)	169

Symmetry codes: (i) 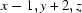; (ii) 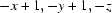; (iii) 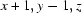.

**Fig. 1.**
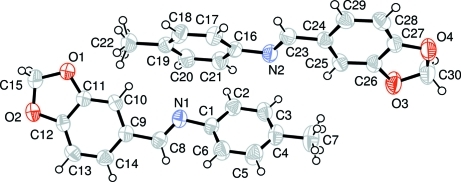
View of the title compound with the atom numbering scheme. The displacement ellipsoids are drawn at the 50% probability level. H-atoms are shown by small circles of arbitrary radii.

**Fig. 2.**
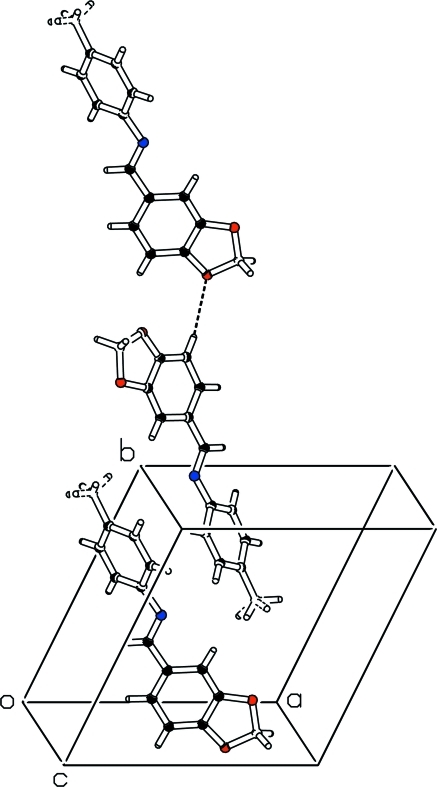
The partial crystal packing (PLATON; Spek, 2009)

## supplementary crystallographic information

### Comment

The title compound (I, Fig. 1) is being reported as a part of our ongoing project related to synthesis of various Schiff bases of piperonal and then their metal complexation (Tahir *et al.*, 2010).

The title compound consists of two molecules in the crystallographic asymmetric unit which differ from each other geometrically. In one molecule, 4-methylanilinic group A (C1—C7/N1) and the piperonalic group B (C8—C15/O1/O2) are almost planar with r. m. s deviation of 0.0511 and 0.0241 Å, respectively. The dihedral angle between A/B is 42.90 (6)°. In second molecule, the 4-methylanilinic group C (C16—C22/N2) and the piperonalic group D (C23—C30/O3/O4) are also almost planar with r. m. s deviation of 0.0082 and 0.0486 Å, respectively. The dihedral angle between C/D is 19.40 (5)°. The molecules are interlinked through hydrogen bonds of C—H···O type (Table 1, Fig. 2). The C—H···π interactions (Table 1) play important role in consolidating the crystal packing. The H atoms of methyl groups are disordered over two set of sites with equal occupancy ratio.

### Experimental

Equimolar quantities of 4-methylaniline and and piperonal were refluxed in methanol along with few drops of acetic acid as catalyst for 30 min resulting in orange yellow solution. The solution was kept at room temperature which affoarded orange yellow prisms after a week.

### Refinement

The H atoms were positioned geometrically (C–H = 0.93–0.97 Å) and were included in the refinement in the riding model approximation, with *U*_iso_(H) = *xU*_eq_(C), where *x* = 1.5 for methyl H-atoms and *x* = 1.2 for aryl H-atoms. The H-atoms of methyl groups are disordered over two set of sites with equal occupancy ratio.

### Crystal data

**Table:**

C_15_H_13_NO_2_	*Z* = 4
*M**_r_* = 239.26	*F*(000) = 504
Triclinic, *P*1	*D*_x_ = 1.295 Mg m^−^^3^
Hall symbol: -P 1	Mo *K*α radiation, λ = 0.71073 Å
*a* = 10.6914 (4) Å	Cell parameters from 3294 reflections
*b* = 10.7680 (3) Å	θ = 2.2–25.3°
*c* = 13.3332 (5) Å	µ = 0.09 mm^−^^1^
α = 89.443 (2)°	*T* = 296 K
β = 67.112 (2)°	Prisms, yellow
γ = 62.534 (1)°	0.32 × 0.22 × 0.18 mm
*V* = 1227.41 (8) Å^3^	

### Data collection

**Table:**

Bruker Kappa APEXII CCD diffractometer	4417 independent reflections
Radiation source: fine-focus sealed tube	3294 reflections with *I* > 2σ(*I*)
graphite	*R*_int_ = 0.026
Detector resolution: 8.20 pixels mm^-1^	θ_max_ = 25.3°, θ_min_ = 2.8°
ω scans	*h* = −11→12
Absorption correction: multi-scan (*SADABS*; Bruker, 2005)	*k* = −12→12
*T*_min_ = 0.980, *T*_max_ = 0.988	*l* = −16→16
18069 measured reflections	

### Refinement

**Table:**

Refinement on *F*^2^	Primary atom site location: structure-invariant direct methods
Least-squares matrix: full	Secondary atom site location: difference Fourier map
*R*[*F*^2^ > 2σ(*F*^2^)] = 0.039	Hydrogen site location: inferred from neighbouring sites
*wR*(*F*^2^) = 0.110	H-atom parameters constrained
*S* = 1.03	*w* = 1/[σ^2^(*F*_o_^2^) + (0.050*P*)^2^ + 0.2009*P*] where *P* = (*F*_o_^2^ + 2*F*_c_^2^)/3
4417 reflections	(Δ/σ)_max_ < 0.001
323 parameters	Δρ_max_ = 0.17 e Å^−^^3^
0 restraints	Δρ_min_ = −0.15 e Å^−^^3^

### Special details

**Table:**

Geometry. Bond distances, angles etc. have been calculated using the rounded fractional coordinates. All su's are estimated from the variances of the (full) variance-covariance matrix. The cell esds are taken into account in the estimation of distances, angles and torsion angles
Refinement. Refinement of *F*^2^ against ALL reflections. The weighted *R*-factor *wR* and goodness of fit *S* are based on *F*^2^, conventional *R*-factors *R* are based on *F*, with *F* set to zero for negative *F*^2^. The threshold expression of *F*^2^ > σ(*F*^2^) is used only for calculating *R*-factors(gt) *etc*. and is not relevant to the choice of reflections for refinement. *R*-factors based on *F*^2^ are statistically about twice as large as those based on *F*, and *R*- factors based on ALL data will be even larger.

### Fractional atomic coordinates and isotropic or equivalent isotropic displacement parameters (Å^2^)

**Table:**

	*x*	*y*	*z*	*U*_iso_*/*U*_eq_	Occ. (<1)
O1	0.85323 (13)	0.05174 (12)	0.16217 (13)	0.0823 (5)	
O2	0.84925 (13)	−0.15617 (11)	0.13086 (10)	0.0649 (4)	
N1	0.27161 (15)	0.46441 (13)	0.35889 (10)	0.0525 (4)	
C1	0.12804 (17)	0.59481 (15)	0.39573 (12)	0.0479 (5)	
C2	0.10114 (18)	0.70402 (16)	0.46908 (13)	0.0545 (5)	
C3	−0.0303 (2)	0.83722 (17)	0.50051 (14)	0.0594 (6)	
C4	−0.1380 (2)	0.86823 (17)	0.45801 (14)	0.0603 (6)	
C5	−0.1111 (2)	0.75802 (19)	0.38577 (16)	0.0702 (7)	
C6	0.0193 (2)	0.62326 (18)	0.35508 (15)	0.0649 (6)	
C7	−0.27620 (18)	1.01681 (13)	0.48639 (16)	0.0868 (8)	
C8	0.27767 (15)	0.34643 (13)	0.33754 (12)	0.0518 (5)	
C9	0.42524 (17)	0.21151 (15)	0.28682 (12)	0.0476 (5)	
C10	0.56824 (18)	0.20906 (15)	0.25598 (13)	0.0527 (5)	
C11	0.70124 (18)	0.08074 (16)	0.20435 (13)	0.0512 (5)	
C12	0.69907 (18)	−0.04387 (14)	0.18398 (12)	0.0481 (5)	
C13	0.56266 (19)	−0.04509 (16)	0.21265 (13)	0.0547 (6)	
C14	0.42526 (19)	0.08612 (16)	0.26411 (13)	0.0534 (6)	
C15	0.9484 (2)	−0.09882 (17)	0.12271 (18)	0.0729 (7)	
O3	−0.35490 (13)	1.45856 (11)	0.39324 (10)	0.0723 (4)	
O4	−0.34615 (13)	1.64734 (11)	0.31303 (10)	0.0668 (4)	
N2	0.18413 (14)	1.00194 (12)	0.16824 (10)	0.0483 (4)	
C16	0.31939 (16)	0.86467 (14)	0.12541 (12)	0.0431 (5)	
C17	0.43932 (17)	0.81718 (16)	0.01782 (12)	0.0513 (5)	
C18	0.56401 (18)	0.67969 (17)	−0.01516 (13)	0.0544 (5)	
C19	0.57678 (17)	0.58422 (15)	0.05568 (13)	0.0499 (5)	
C20	0.45610 (19)	0.63169 (16)	0.16171 (14)	0.0563 (6)	
C21	0.32925 (18)	0.76868 (16)	0.19582 (13)	0.0532 (5)	
C22	0.71353 (14)	0.43394 (12)	0.01711 (12)	0.0656 (6)	
C23	0.18014 (14)	1.10785 (12)	0.12391 (11)	0.0479 (5)	
C24	0.04255 (17)	1.25022 (14)	0.16788 (12)	0.0439 (5)	
C25	−0.09113 (17)	1.27386 (15)	0.26267 (12)	0.0485 (5)	
C26	−0.21271 (17)	1.40978 (15)	0.30257 (12)	0.0486 (5)	
C27	−0.20845 (18)	1.52255 (15)	0.25424 (13)	0.0484 (5)	
C28	−0.08194 (19)	1.50285 (16)	0.16051 (13)	0.0539 (6)	
C29	0.04428 (18)	1.36396 (15)	0.11813 (13)	0.0512 (5)	
C30	−0.4330 (2)	1.61064 (18)	0.40804 (17)	0.0795 (7)	
H2	0.17288	0.68733	0.49767	0.0655*	
H3	−0.04678	0.90784	0.55151	0.0712*	
H5	−0.18260	0.77485	0.35692	0.0843*	
H6	0.03349	0.55134	0.30662	0.0778*	
H7A	−0.29853	1.06796	0.55526	0.1302*	0.500
H7B	−0.36576	1.01018	0.49375	0.1302*	0.500
H7C	−0.25211	1.06673	0.42819	0.1302*	0.500
H7D	−0.31240	1.02862	0.42953	0.1302*	0.500
H7E	−0.24517	1.08640	0.49106	0.1302*	0.500
H7F	−0.35883	1.02984	0.55661	0.1302*	0.500
H8	0.18479	0.34604	0.35484	0.0622*	
H10	0.57163	0.29154	0.27028	0.0632*	
H13	0.56126	−0.12878	0.19867	0.0656*	
H14	0.32993	0.08964	0.28396	0.0640*	
H15A	1.00499	−0.14057	0.16709	0.0875*	
H15B	1.02381	−0.12057	0.04595	0.0875*	
H17	0.43518	0.87851	−0.03190	0.0616*	
H18	0.64236	0.64993	−0.08751	0.0653*	
H20	0.46038	0.57005	0.21120	0.0676*	
H21	0.24912	0.79690	0.26730	0.0638*	
H22A	0.80663	0.43643	0.00573	0.0984*	0.500
H22B	0.72685	0.39048	−0.05150	0.0984*	0.500
H22C	0.69426	0.37915	0.07252	0.0984*	0.500
H22D	0.67853	0.36761	0.01210	0.0984*	0.500
H22E	0.75831	0.41356	0.06933	0.0984*	0.500
H22F	0.79090	0.42489	−0.05468	0.0984*	0.500
H23	0.26796	1.09409	0.06129	0.0575*	
H25	−0.09608	1.19938	0.29667	0.0582*	
H28	−0.08011	1.57831	0.12668	0.0647*	
H29	0.13266	1.34664	0.05446	0.0615*	
H30A	−0.43932	1.65312	0.47490	0.0954*	
H30B	−0.53839	1.64639	0.41559	0.0954*	

### Atomic displacement parameters (Å^2^)

**Table:**

	*U*^11^	*U*^22^	*U*^33^	*U*^12^	*U*^13^	*U*^23^
O1	0.0454 (7)	0.0472 (7)	0.1337 (12)	−0.0163 (6)	−0.0273 (7)	−0.0082 (7)
O2	0.0563 (7)	0.0391 (6)	0.0825 (8)	−0.0145 (6)	−0.0256 (6)	0.0000 (5)
N1	0.0473 (8)	0.0487 (7)	0.0477 (8)	−0.0166 (6)	−0.0165 (6)	0.0037 (6)
C1	0.0435 (9)	0.0477 (8)	0.0422 (8)	−0.0183 (7)	−0.0143 (7)	0.0074 (7)
C2	0.0507 (10)	0.0553 (9)	0.0512 (9)	−0.0237 (8)	−0.0193 (8)	0.0069 (7)
C3	0.0606 (11)	0.0488 (9)	0.0530 (10)	−0.0229 (9)	−0.0151 (9)	0.0045 (7)
C4	0.0546 (10)	0.0490 (9)	0.0536 (10)	−0.0149 (8)	−0.0149 (8)	0.0145 (8)
C5	0.0645 (12)	0.0657 (11)	0.0755 (12)	−0.0200 (10)	−0.0410 (10)	0.0156 (10)
C6	0.0650 (12)	0.0556 (10)	0.0672 (11)	−0.0195 (9)	−0.0350 (10)	0.0022 (8)
C7	0.0789 (14)	0.0574 (11)	0.0812 (14)	−0.0085 (10)	−0.0263 (11)	0.0199 (10)
C8	0.0470 (9)	0.0536 (9)	0.0472 (9)	−0.0224 (8)	−0.0166 (7)	0.0104 (7)
C9	0.0474 (9)	0.0462 (8)	0.0435 (8)	−0.0203 (7)	−0.0181 (7)	0.0101 (6)
C10	0.0530 (10)	0.0398 (8)	0.0610 (10)	−0.0210 (8)	−0.0232 (8)	0.0034 (7)
C11	0.0450 (9)	0.0452 (8)	0.0600 (10)	−0.0202 (8)	−0.0222 (8)	0.0052 (7)
C12	0.0513 (10)	0.0371 (8)	0.0489 (9)	−0.0164 (7)	−0.0221 (7)	0.0080 (6)
C13	0.0666 (11)	0.0447 (8)	0.0590 (10)	−0.0321 (8)	−0.0276 (9)	0.0141 (7)
C14	0.0527 (10)	0.0549 (9)	0.0563 (10)	−0.0311 (8)	−0.0219 (8)	0.0166 (7)
C15	0.0519 (11)	0.0451 (9)	0.0973 (14)	−0.0146 (9)	−0.0212 (10)	0.0035 (9)
O3	0.0521 (7)	0.0471 (6)	0.0740 (8)	−0.0125 (6)	−0.0016 (6)	0.0077 (6)
O4	0.0593 (7)	0.0405 (6)	0.0775 (8)	−0.0121 (6)	−0.0237 (6)	0.0094 (5)
N2	0.0434 (7)	0.0425 (7)	0.0483 (7)	−0.0166 (6)	−0.0156 (6)	0.0054 (5)
C16	0.0385 (8)	0.0420 (8)	0.0451 (8)	−0.0178 (7)	−0.0172 (7)	0.0047 (6)
C17	0.0473 (9)	0.0499 (9)	0.0474 (9)	−0.0192 (8)	−0.0179 (8)	0.0118 (7)
C18	0.0413 (9)	0.0562 (9)	0.0484 (9)	−0.0171 (8)	−0.0119 (7)	0.0019 (7)
C19	0.0421 (9)	0.0448 (8)	0.0634 (10)	−0.0189 (7)	−0.0267 (8)	0.0049 (7)
C20	0.0604 (11)	0.0469 (9)	0.0606 (10)	−0.0241 (8)	−0.0285 (9)	0.0165 (8)
C21	0.0519 (10)	0.0502 (9)	0.0472 (9)	−0.0233 (8)	−0.0144 (8)	0.0089 (7)
C22	0.0526 (10)	0.0481 (9)	0.0890 (13)	−0.0156 (8)	−0.0355 (10)	0.0026 (8)
C23	0.0456 (9)	0.0484 (8)	0.0440 (8)	−0.0234 (7)	−0.0139 (7)	0.0039 (7)
C24	0.0464 (9)	0.0421 (8)	0.0430 (8)	−0.0214 (7)	−0.0197 (7)	0.0053 (6)
C25	0.0528 (9)	0.0399 (8)	0.0480 (9)	−0.0223 (7)	−0.0182 (8)	0.0091 (6)
C26	0.0445 (9)	0.0436 (8)	0.0481 (9)	−0.0186 (7)	−0.0151 (7)	0.0049 (7)
C27	0.0502 (9)	0.0380 (8)	0.0562 (9)	−0.0179 (7)	−0.0272 (8)	0.0072 (7)
C28	0.0661 (11)	0.0472 (9)	0.0588 (10)	−0.0325 (8)	−0.0315 (9)	0.0190 (7)
C29	0.0535 (10)	0.0519 (9)	0.0485 (9)	−0.0302 (8)	−0.0173 (8)	0.0101 (7)
C30	0.0655 (12)	0.0465 (10)	0.0794 (13)	−0.0093 (9)	−0.0105 (10)	0.0069 (9)

### Geometric parameters (Å, °)

**Table:**

O1—C11	1.371 (3)	C8—H8	0.9300
O1—C15	1.421 (2)	C10—H10	0.9300
O2—C12	1.372 (2)	C13—H13	0.9300
O2—C15	1.425 (3)	C14—H14	0.9300
O3—C26	1.377 (2)	C15—H15B	0.9700
O3—C30	1.426 (2)	C15—H15A	0.9700
O4—C27	1.373 (2)	C16—C17	1.394 (2)
O4—C30	1.427 (2)	C16—C21	1.385 (2)
N1—C1	1.417 (2)	C17—C18	1.378 (2)
N1—C8	1.271 (2)	C18—C19	1.386 (2)
N2—C16	1.416 (2)	C19—C20	1.383 (2)
N2—C23	1.2702 (17)	C19—C22	1.507 (2)
C1—C2	1.383 (2)	C20—C21	1.382 (2)
C1—C6	1.378 (3)	C23—C24	1.458 (2)
C2—C3	1.379 (2)	C24—C25	1.404 (2)
C3—C4	1.381 (3)	C24—C29	1.391 (2)
C4—C5	1.382 (3)	C25—C26	1.355 (2)
C4—C7	1.511 (2)	C26—C27	1.380 (2)
C5—C6	1.385 (3)	C27—C28	1.365 (3)
C8—C9	1.460 (2)	C28—C29	1.392 (2)
C9—C10	1.408 (3)	C17—H17	0.9300
C9—C14	1.387 (2)	C18—H18	0.9300
C10—C11	1.360 (2)	C20—H20	0.9300
C11—C12	1.384 (2)	C21—H21	0.9300
C12—C13	1.363 (3)	C22—H22A	0.9600
C13—C14	1.395 (2)	C22—H22B	0.9600
C2—H2	0.9300	C22—H22C	0.9600
C3—H3	0.9300	C22—H22D	0.9600
C5—H5	0.9300	C22—H22E	0.9600
C6—H6	0.9300	C22—H22F	0.9600
C7—H7D	0.9600	C23—H23	0.9300
C7—H7E	0.9600	C25—H25	0.9300
C7—H7A	0.9600	C28—H28	0.9300
C7—H7B	0.9600	C29—H29	0.9300
C7—H7C	0.9600	C30—H30A	0.9700
C7—H7F	0.9600	C30—H30B	0.9700
			
C11—O1—C15	106.38 (15)	O2—C15—H15A	110.00
C12—O2—C15	105.90 (13)	O2—C15—H15B	110.00
C26—O3—C30	105.62 (14)	O1—C15—H15A	110.00
C27—O4—C30	105.60 (12)	N2—C16—C17	125.51 (13)
C1—N1—C8	120.32 (17)	N2—C16—C21	116.66 (14)
C16—N2—C23	121.55 (14)	C17—C16—C21	117.79 (14)
N1—C1—C2	118.47 (17)	C16—C17—C18	120.24 (14)
N1—C1—C6	123.39 (15)	C17—C18—C19	122.32 (15)
C2—C1—C6	117.89 (16)	C18—C19—C20	116.99 (15)
C1—C2—C3	121.09 (19)	C18—C19—C22	121.24 (14)
C2—C3—C4	121.56 (17)	C20—C19—C22	121.74 (14)
C3—C4—C5	116.92 (17)	C19—C20—C21	121.48 (15)
C5—C4—C7	121.5 (2)	C16—C21—C20	121.15 (15)
C3—C4—C7	121.57 (17)	N2—C23—C24	122.21 (14)
C4—C5—C6	121.9 (2)	C23—C24—C25	119.96 (13)
C1—C6—C5	120.59 (18)	C23—C24—C29	120.34 (15)
N1—C8—C9	122.35 (17)	C25—C24—C29	119.68 (14)
C8—C9—C10	120.28 (15)	C24—C25—C26	117.21 (14)
C8—C9—C14	119.97 (18)	O3—C26—C25	127.71 (14)
C10—C9—C14	119.68 (15)	O3—C26—C27	109.61 (13)
C9—C10—C11	117.06 (16)	C25—C26—C27	122.69 (16)
O1—C11—C12	109.29 (15)	O4—C27—C26	109.96 (15)
O1—C11—C10	128.18 (17)	O4—C27—C28	128.39 (14)
C10—C11—C12	122.5 (2)	C26—C27—C28	121.64 (15)
O2—C12—C11	109.89 (17)	C27—C28—C29	116.57 (15)
O2—C12—C13	128.21 (15)	C24—C29—C28	122.18 (16)
C11—C12—C13	121.88 (15)	O3—C30—O4	108.15 (15)
C12—C13—C14	116.30 (17)	C16—C17—H17	120.00
C9—C14—C13	122.6 (2)	C18—C17—H17	120.00
O1—C15—O2	108.12 (17)	C17—C18—H18	119.00
C1—C2—H2	119.00	C19—C18—H18	119.00
C3—C2—H2	119.00	C19—C20—H20	119.00
C2—C3—H3	119.00	C21—C20—H20	119.00
C4—C3—H3	119.00	C16—C21—H21	119.00
C4—C5—H5	119.00	C20—C21—H21	119.00
C6—C5—H5	119.00	C19—C22—H22A	109.00
C5—C6—H6	120.00	C19—C22—H22B	109.00
C1—C6—H6	120.00	C19—C22—H22C	109.00
C4—C7—H7A	109.00	C19—C22—H22D	109.00
C4—C7—H7B	109.00	C19—C22—H22E	109.00
C4—C7—H7C	109.00	C19—C22—H22F	109.00
H7A—C7—H7B	109.00	H22A—C22—H22B	109.00
H7A—C7—H7C	109.00	H22A—C22—H22C	109.00
H7A—C7—H7D	141.00	H22A—C22—H22D	141.00
H7A—C7—H7E	56.00	H22A—C22—H22E	56.00
H7A—C7—H7F	56.00	H22A—C22—H22F	56.00
H7B—C7—H7C	109.00	H22B—C22—H22C	109.00
H7B—C7—H7D	56.00	H22B—C22—H22D	56.00
H7B—C7—H7E	141.00	H22B—C22—H22E	141.00
H7B—C7—H7F	56.00	H22B—C22—H22F	56.00
H7C—C7—H7D	56.00	H22C—C22—H22D	56.00
H7C—C7—H7E	56.00	H22C—C22—H22E	56.00
H7C—C7—H7F	141.00	H22C—C22—H22F	141.00
H7D—C7—H7E	109.00	H22D—C22—H22E	109.00
H7D—C7—H7F	109.00	H22D—C22—H22F	109.00
H7E—C7—H7F	109.00	H22E—C22—H22F	109.00
C4—C7—H7F	109.00	N2—C23—H23	119.00
C4—C7—H7E	109.00	C24—C23—H23	119.00
C4—C7—H7D	109.00	C24—C25—H25	121.00
N1—C8—H8	119.00	C26—C25—H25	121.00
C9—C8—H8	119.00	C27—C28—H28	122.00
C9—C10—H10	121.00	C29—C28—H28	122.00
C11—C10—H10	121.00	C24—C29—H29	119.00
C12—C13—H13	122.00	C28—C29—H29	119.00
C14—C13—H13	122.00	O3—C30—H30A	110.00
C9—C14—H14	119.00	O3—C30—H30B	110.00
C13—C14—H14	119.00	O4—C30—H30A	110.00
O1—C15—H15B	110.00	O4—C30—H30B	110.00
H15A—C15—H15B	108.00	H30A—C30—H30B	108.00
			
C15—O1—C11—C10	176.76 (18)	C9—C10—C11—O1	176.83 (16)
C15—O1—C11—C12	−5.02 (19)	C9—C10—C11—C12	−1.2 (2)
C11—O1—C15—O2	6.6 (2)	C10—C11—C12—O2	179.84 (15)
C15—O2—C12—C11	2.67 (18)	C10—C11—C12—C13	1.3 (3)
C15—O2—C12—C13	−178.93 (17)	O1—C11—C12—C13	−177.02 (15)
C12—O2—C15—O1	−5.73 (19)	O1—C11—C12—O2	1.50 (18)
C26—O3—C30—O4	10.3 (2)	O2—C12—C13—C14	−178.49 (15)
C30—O3—C26—C27	−7.0 (2)	C11—C12—C13—C14	−0.3 (2)
C30—O3—C26—C25	173.0 (2)	C12—C13—C14—C9	−0.9 (2)
C27—O4—C30—O3	−9.8 (2)	N2—C16—C17—C18	−178.61 (19)
C30—O4—C27—C28	−175.6 (2)	C21—C16—C17—C18	−1.2 (3)
C30—O4—C27—C26	5.5 (2)	N2—C16—C21—C20	179.60 (19)
C1—N1—C8—C9	172.46 (13)	C17—C16—C21—C20	1.9 (3)
C8—N1—C1—C6	−38.5 (2)	C16—C17—C18—C19	−0.5 (3)
C8—N1—C1—C2	147.31 (16)	C17—C18—C19—C20	1.4 (3)
C16—N2—C23—C24	−179.17 (16)	C17—C18—C19—C22	179.34 (19)
C23—N2—C16—C21	161.80 (18)	C18—C19—C20—C21	−0.6 (3)
C23—N2—C16—C17	−20.7 (3)	C22—C19—C20—C21	−178.55 (19)
N1—C1—C2—C3	174.47 (15)	C19—C20—C21—C16	−1.0 (3)
C2—C1—C6—C5	1.1 (3)	N2—C23—C24—C25	0.4 (3)
N1—C1—C6—C5	−173.16 (17)	N2—C23—C24—C29	178.66 (17)
C6—C1—C2—C3	0.0 (3)	C23—C24—C25—C26	177.07 (17)
C1—C2—C3—C4	−1.8 (3)	C29—C24—C25—C26	−1.2 (3)
C2—C3—C4—C7	−175.81 (17)	C23—C24—C29—C28	−176.98 (18)
C2—C3—C4—C5	2.4 (3)	C25—C24—C29—C28	1.3 (3)
C3—C4—C5—C6	−1.4 (3)	C24—C25—C26—O3	179.66 (18)
C7—C4—C5—C6	176.84 (18)	C24—C25—C26—C27	−0.4 (3)
C4—C5—C6—C1	−0.3 (3)	O3—C26—C27—O4	1.0 (2)
N1—C8—C9—C14	−179.24 (15)	O3—C26—C27—C28	−178.02 (18)
N1—C8—C9—C10	−2.3 (2)	C25—C26—C27—O4	−179.03 (17)
C8—C9—C10—C11	−176.93 (14)	C25—C26—C27—C28	2.0 (3)
C14—C9—C10—C11	0.1 (2)	O4—C27—C28—C29	179.37 (19)
C10—C9—C14—C13	1.0 (2)	C26—C27—C28—C29	−1.9 (3)
C8—C9—C14—C13	177.97 (15)	C27—C28—C29—C24	0.2 (3)

### Hydrogen-bond geometry (Å, °)

**Table:**

Cg3, Cg5 and Cg6 are the centroids of the C9–C14, C16–C21 and C24–C29 rings, respectively.

**Table:**

*D*—H···*A*	*D*—H	H···*A*	*D*···*A*	*D*—H···*A*
C28—H28···O2^i^	0.93	2.59	3.447 (2)	153
C17—H17···Cg3^ii^	0.93	2.73	3.5306 (16)	145
C22—H22B···Cg5^ii^	0.96	2.86	3.5480 (18)	130
C22—H22D···Cg5^ii^	0.96	2.71	3.5480 (18)	146
C22—H22E···Cg6^iii^	0.96	2.91	3.8610 (18)	169

Symmetry codes: (i) *x*−1, *y*+2, *z*; (ii) −*x*+1, −*y*+1, −*z*; (iii) *x*+1, *y*−1, *z*.
